# The Use of Meta-Analysis and Forest Plots to Examine and Display Data in Oncology Research

**Published:** 2014-11-01

**Authors:** Joanne Lester

**Affiliations:** The Ohio State University, Columbus, Ohio

In the article authored by Thomas et al. (2014) discussed by Carolyn Grande and Marcia Brose on page 461, there are several statistical tools used to understand the data. The data are presented in a *quantitative* fashion, e.g., numerical measures of outcomes. The statistical tool of *meta-analysis* is used to quantitatively answer the authors’ research question: "What is the effect of sorafenib on metastatic thyroid cancer in mixed histologic groups?" In order to visualize the quantitative results of this meta-analysis, a *forest plot* is used. This forest plot displays summarized quantitative data about each study (e.g., the meta-analysis) and an estimated overall quantitative value for the combined studies. These statistical methods represent some of the highest and most trusted methods of data representation. Read on to learn more about meta-analysis and forest plots. To view multiple examples of forest plots, visit https://www.statsdirect.com/help/graphics/cochrane_plot.htm.

## META-ANALYSIS

A meta-analysis is a form of quantitative statistics that is applied to separate but similar studies that are typically performed by other researchers. A meta-analysis can provide robust data and is the highest level of evidence about a stated topic. All relevant publications within a given time period are reviewed, summarized, and measured (Schriger, Altman, Vetter, Heafner, & Moher, 2010). Inclusion and exclusion criteria are outlined to guide the study reviews and seek data related to the stated research question. Data from the individual studies are summarized and then pooled and calculated to provide an overall estimate of study outcomes. The resulting data are presented in a graph, most commonly a forest plot.

Data outcomes are often used to illustrate gaps in the literature and subsequently guide future clinical trials, or may provide robust data to be incorporated into clinical practice. In the article by Thomas et al. (2014), all data relevant to the research question were sought to compare the efficacy of sorafenib in various histologic types of radioiodine-resistant metastatic thyroid cancers.

For the Thomas et al. meta-analysis, a systematic electronic review of completed studies was conducted within an identified time period (before December 2012), in specified databases (PubMed, Embase, Medline) and with stated search terms (thyroid cancer and sorafenib; Thomas et al., 2014). In addition, these authors manually searched the bibliographies from identified studies for additional information; authors of selected trials were contacted for additional details or updates on their studies (Thomas et al., 2014).

## INCLUSION CRITERIA AND METHODS

Inclusion criteria included English language, adult patients, identified response rates, variable histology, treatment with single-agent sorafenib at 400 mg twice daily, and standard reporting criteria for response and adverse events. Exclusion criteria included nonthyroid cancers, studies using multiple drugs, case reports, review articles, and phase I studies. Additional criteria included response rate (RR), adverse events (AEs), and median progression-free survival (PFS), as associated with sorafenib (Thomas et al., 2014).

The electronic search identified nine studies for review, although only eight were used in the meta-analysis as one study had a different drug dosing than what was cited in the inclusion criteria. Combined, the studies included 219 subjects with variable histology: papillary, follicular, or poorly differentiated (n = 159), metastatic (n = 52), and anaplastic (n = 8) thyroid cancers. Each study was weighted by sample size; two studies were combined and weighted as one due to the length of follow-up. Therefore, eight studies were reviewed and reported, although it would be reported as seven. Quantitative data such as response rates, adverse events, median time of progression-free survival, and 95% confidence interval were estimated in order to evaluate all studies, centered on the same criteria (Thomas et al., 2014).

All eight of the studies utilized RECIST v.1.0 (Response Evaluation in Solid Tumors) for evaluation of side effects; seven of the eight studies also used CTCAE v.3 (Common Terminology Criteria for Adverse Events). Of the publications, six were prospective phase II trials; two were retrospective studies. Three independent reviewers were used to tabulate data; no specific tools were used. The following statistical software programs were used for all analyses: SAS version 9.2 (SAS Institute, Inc., Cary, NC, http://www.sas.com) and S-plus (TIBCO Software Inc., Palo Alto, CA, http://www.tibco.com; Thomas et al., 2014).

Summarily, this meta-analysis of patients (N = 219) treated with sorafenib for metastatic thyroid cancers demonstrated the following response rates: partial (21%), and stable (60%) and progressive disease (20%), or 81% of the patients treated with sorafenib had a partial response or stable disease. Complete response rates were not reported. Histologically, medullary thyroid cancer had the best partial response rate, followed by differentiated thyroid cancer; low response rates were observed in those patients with anaplastic thyroid cancers. Regardless of histology, the overall median progression-free survival rate was 18 months (Thomas et al., 2014), which are the data displayed in the forest plot.

## FOREST PLOTS

A forest plot was used to display median progression-free survival for the seven studies. The solid lines represent the 95% confidence interval, e.g., the chance that these results would occur in 95% of cases. Two studies from the meta-analysis had no data related to the 95% confidence interval; in two other studies the upper limit of the 95% confidence interval was not reached. A faint red diamond indicates overall median progression-free survival of 17.9 months (95% CI = 17.9–18; Thomas et al., 2014).

**Elements**

The term forest plot refers to the forest of lines that are used to represent multiple individual studies plotted against the same axis, e.g., confidence interval. Forest plots are graphic displays that are used to illustrate individual and estimated group data from a meta-analysis of multiple quantitative studies that answer the same research question (Schriger et al., 2010). Forest plots are typically represented with a vertical axis (top to bottom) and horizontal axis (right to left) with standard descriptions due to the convenience of statistical programs (Schriger et al., 2010). This provides a one-square visual glance of individual study heterogeneity and overall effect estimates (Schriger et al., 2010).

*Horizontal axis*: Each study is represented as a horizontal line on the y-axis to list the primary author and year of study, characteristics, number of participants in treatment and control arms, outcome measure, relative risk based on numerical confidence interval (e.g., breadth of sample in 95% CI), and percent of weight (Schriger et al., 2010). Thomas et al. (2014) list only the primary author’s name. Plots are typically ordered with the least achieving studies at the top and the best studies toward the bottom of the graph, which is evident in the Thomas et al. (2014) article.

The confidence interval for each study is represented by a horizontal solid line centered on the vertical CI line. This provides a visual marker that indicates the study’s mean results within the study’s stated confidence intervals. Thomas et al. (2014) included the confidence interval for the three studies that did include it. In the four studies that did not have a stated confidence interval, the mean outcome is represented by a dot on the graph, with the accompanying author’s name on the left border of the graph.

*Vertical axis*: The vertical axis describes information in the columns that coincide with the horizontal data (Schriger et al., 2010). The vertical axis headings include name of study/author, characteristics of study, confidence interval (direction of effect), treatment acceptance (optional), numerical response rate of CI, sample sizes, and influence of each study on overall estimate. The referenced table in Thomas et al. (2014) does not include these items: The area is left blank. A solid vertical line represents the confidence interval accepted for the total study. The positive effect is to the left of the vertical line, and the negative effect is to the right. In the Thomas et al. (2014) table, an imaginary line must be drawn upward from the "ideal" outcomes which is represented by a red diamond at the bottom of the graph. The point estimate of each study is plotted on this axis (Steff & Clarke, 2001). In the event that the confidence interval is not reached by an individual study, a dot alone may be used to indicate the level of data, as noted in the Thomas et al. (2014) table. The wider the horizontal line, the less precise is the representation of data in relation to the overall study; a wider line indicates a larger spread of the study’s confidence interval.

Forest plots are considered a powerful tool to represent meta-analyses (Schriger et al., 2010). Forest plots are commonly used to illustrate clinical study results although should not be used to represent large groups of studies, or large amounts of data as the graph may be unwieldy. It is *important* to read and understand the parameters of the meta-analysis prior to making a decision about the study based upon data alone on the plot. These factors about the Thomas et al. (2014) are clearly explained in article by Grande and Brose. The plot may represent a high response rate, but not include measures about significant side effects, or dose reductions secondary to adverse events.

*Number of participants*: The left-or right-hand margin of the forest plot often includes the number (N) of participants of each individual study in the meta-analysis (Schriger et al., 2010). The number of treatment participants (n) is noted with the total number (N) of study (e.g., 99/100). When the study is a randomized study with a placebo arm, there are often two columns in the left- or right-hand margin: one that denotes participants on treatment arm of study (e.g., 89/100), and one that denotes those on control arm of study (e.g., 45/50). The table in Thomas et al. (2014) does not include these items.

*Confidence interval*: The confidence interval (CI) of clinical trial results is stated in each study as a measure of the reliability (e.g., that the study is repeatable over and over with nearly the same results) of the study (Schriger et al., 2010). In scientific clinical studies, the most common CI is 95%, although it may be 99% when the chance of error is unacceptable. The confidence interval indicates the confidence of the researcher that their results are correct (e.g., 95%) and the risk they are willing to take that the remainder (5%) may be incorrect for whatever reason.

In forest plots, the confidence interval is a primary component of the graph; it is designated by a solid vertical line. In the right-hand column of the forest plot, the confidence interval is indicated, with a notation of the relative reliability of individual study results. The confidence intervals of subgroups, e.g., each study, are always wider than the combined confidence interval for the main effect due to smaller numbers (Cuzick, 2005). To resolve this discrepancy, the researcher can perform a test of heterogeneity that determines whether or not a subgroup varies significantly from the estimated main effect (Cuzick, 2005).

*Overall measure of effect*: The overall measure of effect as noted in the meta-analysis is typically represented in dashed lines, or as a red (or black) diamond as noted in the Thomas et al. (2014) table. The center of the diamond represents the overall estimate, and the width or lateral points of the diamond indicate overall confidence intervals (Schriger et al., 2010). The pooled estimate is shown as a number. An additional vertical line is sometimes added to the forest plot to indicate the threshold for clinical relevance, e.g., the effect that is large enough to justify the cost, risks, and inconvenience of the intervention.

*Statistical significance*: The statistical significance of the meta-analysis occurs when the review of multiple eligible studies yields a precise overall estimate with a narrow confidence interval of the pooled estimate. This illustrates the ability of the meta-analysis to significantly answer the research question. Conversely, when the confidence interval includes the vertical line of "no effect," then the result of the meta-analysis is not statistically significant. Of note, while the overall study results may appear significant, it is important to read the text of the article to ensure this significance was earned without a high degree of adverse events or side effects.

**Application in Study of Sorafenib and Thyroid Cancer**

In the study by Thomas et al. (2014) discussed by Grande and Brose, the forest plot is presented in a simple format, centering on the significance of each individual study, and the pooled estimate for progression-free survival. On the horizontal axis, the following information is noted: author of study, median progression-free survival in months, and confidence interval. The vertical columns are titled only with the primary author.

*Horizontal axis*: The seven studies are represented on their respective horizontal lines (two of the original eight eligible studies were combined) using the primary author’s name. Absent are the year of the study, number of participants in treatment and control arms, outcome measure, relative risk of numerical confidence interval, and percent of weight (Thomas et al., 2014). The plots are somewhat ordered with the least achieving studies at the top, and the best studies toward the bottom of the graph. In addition, the overall graph has negative information on the left of the vertical axis, as opposed to the right of the axis. These reversed directions could be confusing to some.

A descriptive summary of the seven studies is presented in Table 1 of the Thomas et al. (2014) article, and includes the total number of patients in each study and the number of patients by distribution of tumor histology. The meta-analysis of response rates to sorafenib based on histologic types of thyroid cancers is noted separately in Table 2. Each of the seven studies is described by number of patients in three categories (partial response, stable disease, and progressive disease) and further subdivided by histologic type (differentiated, metastatic, and anaplastic thyroid cancers). The meta-analysis of adverse event rates is outlined in Table 3. Each adverse event is listed (hand-foot syndrome, diarrhea, skin rash, fatigue, arthralgia/myalgia, weight loss, hypertension, mucositis, hoarseness, dry mouth, and death). The symptoms are further delineated for each study by overall and severe adverse events. The information from Table 3 is not included in the forest plot.

*Confidence interval*: Information about the confidence interval was not provided in the studies by Ahmed et al. or Gupta-Abramson et al. (Thomas et al., 2014). The Ahmed study is inappropriately listed at the top of the graph, given its positive outcome, although perhaps the placement is because of the low number of metastatic patients (n = 1/31). The Lam et al. and Cabanillas et al. studies did not reach the upper limit of the CI for median progression-free survival (Thomas et al., 2014); therefore, they are represented by a dot.

Confidence intervals of each study are represented by a horizontal line, although there is no vertical CI line to assist visual interpretation. One must visually create the vertical line by looking at the center of the overall results diamond (located at the bottom of the graph). The numerical relative risks of the CI per study are not indicated on the graph, although the studies in regard to the primary outcome of the meta-analysis (median progression-free survival) are indicated in 5-month increments (0 to 30 months).

The positive and negative results of the Thomas et al. (2014) meta-analysis are reversed, as compared to the standard presentation of positive results on the left of the vertical axis and negative results on the right (Steff & Clarke, 2001; Schriger et al., 2010). Perhaps the authors provided this presentation as the results of the meta-analysis directly corresponds to progression-free survival (with the exception of Ahmed et al.), as noted at the bottom of the graph, from least to best. Typically, per routine format, the progression-free survival markings would be reversed, e.g., the better responses to the left of the line, and negative responses on the right. Another alternative would be to draw the confidence interval in a horizontal manner, and exhibit negative findings above the line, and positive findings below the line.

*Vertical axis*: The only information that is indicated from the vertical axis is the heading for median progression-free survival in months. There are no headings for the study/author, characteristics of study, confidence interval, treatment acceptance, numerical CI, sample size, or influence of study on overall estimate. The typical solid line indicating accepted CI of the meta-analyses is absent. As described above, the positive and negative results are reversed from the typical presentation. The point estimates are plotted on the imaginary vertical axis. Dots are appropriately used for studies that did not state their CI for progression-free survival (Ahmed et al. and Gupta-Abramson et al.) or for those studies that did not reach their upper CI level (Lam et al. and Cabanillas et al.).

## SUMMARY

Results can be conferred by this forest plot, but it must be closely read and deciphered due to deviations from the norm as described above (Steff & Clarke, 2001; Schriger et al., 2010). The reader must review Tables 1 and 2 to fully understand the forest plot. The significance of adverse events is noted in Table 3. The estimated pooled median for overall progression-free survival is noted by the diamond at the bottom of the graph, indicating a confidence interval of 17.9 months (95% CI = 17.9–18 months). This information is found in the legend of the table instead of in the table itself. All seven of the studies demonstrated a degree of progression-free survival, although they are presented in reverse order.
